# Episcopal Hospital

**Published:** 1861-05

**Authors:** 


EPISCOPAL HOSPITAL.—Few of the charitable institutions of this city can show a more satisfactory career of usefulness than the Hospital of the Protestant Episcopal Church. We learn from the last annual report that the corner-stone of the new building was laid a year ago, and that the work has been pushed rapidly forward. One wing will probably be ready for patients by the close of the present year, and we congratulate the medical staff upon the prospect of being removed from their present limited accommodations, where, for the past nine years, they have labored for the relief of the sick under so many disadvantages.

The outline of the new building was suggested by the celebrated Hôpital Lariboisiere, in which the conveniences and advantages of parallel pavilions are clearly demonstrated. There will be three pavilions, known as the centre building and wings, connected by covered corridors. On the south side of the corridors will be broad verandas. The entire frontage will be 258 feet; the depth of the centre building, including the chapel, 256 feet; the depth of each wing, 200 feet. There will be 63 feet of space between each wing and the chapel.

The rear portions of the wings will be two stories high, and, with the attics, are for wards. The principal wards will be 110 feet long and 30 feet wide, and contain 30 beds each. The attics will contain 15 beds each.

The front portions of the wings will be three stories high, and are principally intended for the domestic purposes of the establishment. There will, however, be on the first and second floors two small wards, and in the basement seven separate rooms for cases of mania a potu, and other exceptional cases. The front of the centre building will be occupied by offices, library, and rooms for twenty-five patients. Thus the whole building will accommodate over two hundred beds. The centre building will contain the operating room, having ample accommodations for students. In the rear of the centre is the chapel. The whole will be heated by steam from an adjoining building, well lighted, abundantly supplied with water, and freely ventilated.

The walls are of brown stone, except the chapel, which is gray, yet harmonizing very well with the other; and the building is of a modified Norman style, and almost fire-proof.

During the past year there were admitted and treated 372 patients; making, with 31 remaining from the previous year, 403 cases.

Of these, 252 recovered, 86 were relieved, 30 died, and 35 remain; 44 were pay, 294 charity; 65 were recent accidents, which are always received in the free beds, yet not considered charity cases; 291 males, 112 females; 202 Protestants, and 201 Romanists.

During the year, 5139 out cases were treated. Since the house has been opened, 2481 cases have been admitted, of which 2121 have been free, and 19,214 out cases have been treated in the same time.

These statistics clearly prove that, while the hospital may be denominational in its title, nothing could be more catholic than the dispensation of its charity.

All suitable applicants, without regard to creed, color, or country, are received, the number being limited only by the capacity of the house. Of incurable cases, of course, preference is given to members of the denomination supporting the establishment. Clergymen of all sects visit the wards at all times, and administer religious consolation according to the peculiar views of the patients.

It reflects great credit upon Dr. Wilson Jewell, the eminent sanitarian, who was the first to urge upon our citizens the necessity of hospital facilities in that locality, though not in connection with the body that has so energetically carried it into operation.

The medical staff consists of Drs. Wm. Mayburry, H. Hartshorne, J. C. Morris, and J. Da Costa; the surgical, of H. E. Drayton, Wm. Hunt, R. S. Thomas, and R. S. Kenderdine.

